# Supplementary material for: Exploring the associations between number of children, multi-partner fertility and risk of obesity at midlife: Findings from the 1970 British Cohort Study (BCS70)
Source: PLoS One. 2023 Apr 13;18(4):e0282795. doi: 10.1371/journal.pone.0282795 (PMC10101483; doi:10.1371/journal.pone.0282795)
Supplement: S1 File — A directed acyclic graph illustrating the relationship between MPF at age 42 and obesity at age 46. (DOCX) [file pone.0282795.s001.docx]

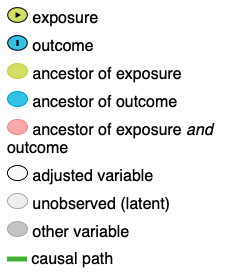
**Supplementary Materials 1. A directed acyclic gra
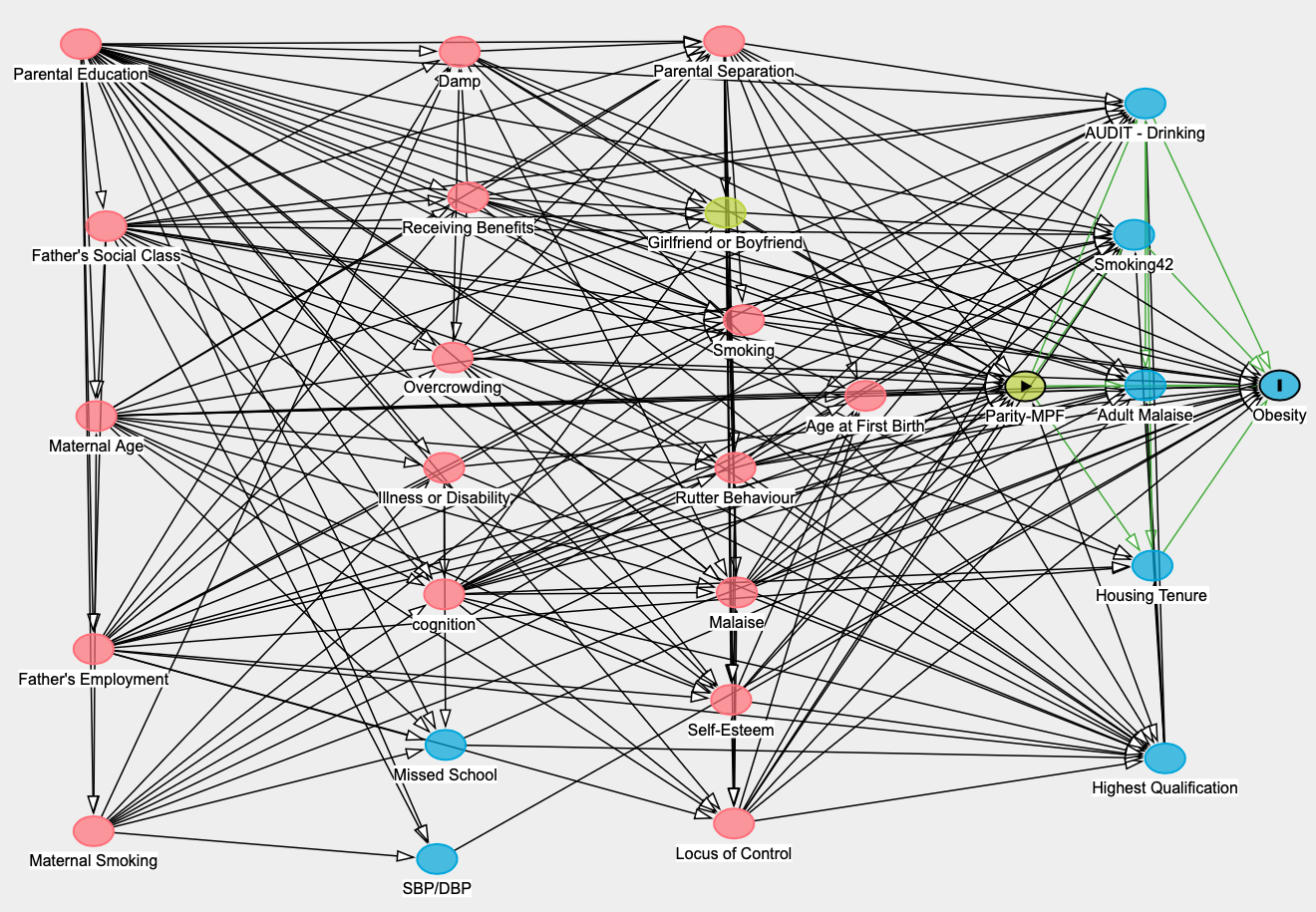
ph illustrating the relationship between MPF at age 42 and obesity at age 46.**

DAGs are a graphical model that depict a set of hypotheses about the causal processes that generate a set of variables of interest. An arrow *X* → *Y* is drawn if there is a hypothesised direct causal effect of *X* on *Y*. An arrow *X* → *Y* only represents that part of the causal effect which is *not* mediated by any of the other variables in the diagram. For pathways where there is certainty that *X* does not have a direct causal influence on *Y*, then the arrow is omitted. A hypothesised causal pathway can also operate through mediators (*X*→*M* →*Y*) if these causal paths start at the exposure, contain only arrows pointing away from the exposure (i.e., to mediators recorded after the exposure), and end at the outcom
