# Supplementary material for: Exploring the associations between number of children, multi-partner fertility and risk of obesity at midlife: Findings from the 1970 British Cohort Study (BCS70)
Source: PLoS One. 2023 Apr 13;18(4):e0282795. doi: 10.1371/journal.pone.0282795 (PMC10101483; doi:10.1371/journal.pone.0282795)
Supplement: S2 File — Level of missing for each variable in the final analytical sample, for fathers and mothers. (DOCX) [file pone.0282795.s002.docx]

**Supplementary Materials 2. Level of missing for each variable in the final analytical sample, for fathers and mothers.**

|  | Fathers (n=2940) | | Mothers (n=3369) | |
| --- | --- | --- | --- | --- |
|  | Number missing | Percentage missing | Number missing | Percentage missing |
| Parental education | 14 | 0.5% | 25 | 0.7% |
| Father’s occupational social class | 8 | 0.3% | 22 | 0.7% |
| Maternal age | 24 | 0.8% | 11 | 0.3% |
| Maternal smoking | 20 | 0.7% | 17 | 0.5% |
| Cognitive ability | 610 | 20.7% | 670 | 19.9% |
| Damp | 372 | 12.7% | 397 | 11.8% |
| Receiving benefits | 892 | 30.3% | 978 | 29.0% |
| Overcrowding | 199 | 6.8% | 228 | 6.8% |
| Illness or disability | 428 | 14.6% | 475 | 14.1% |
| Self-esteem | 2008 | 68.3% | 1947 | 57.8% |
| Locus of control | 2144 | 72.9% | 2075 | 61.6% |
| Parental separation | 1165 | 39.6% | 1168 | 34.7% |
| Smoking | 1832 | 62.3% | 1617 | 48.0% |
| Behaviour | 1398 | 47.6% | 1403 | 41.6% |
| Malaise Index | 684 | 23.3% | 1797 | 53.3% |
| Age at first birth | 482 | 16.4% | 356 | 10.6% |
| Smoking status | 335 | 11.4% | 279 | 8.0% |
| AUDIT – drinking | 783 | 26.6% | 796 | 23.6% |
| Malaise | 684 | 23.3% | 572 | 16.7% |
| Housing tenure | 343 | 11.7% | 274 | 8.1% |
| Highest qualification | 328 | 11.2% | 264 | 7.8% |
