# Supplementary material for: Exploring the associations between number of children, multi-partner fertility and risk of obesity at midlife: Findings from the 1970 British Cohort Study (BCS70)
Source: PLoS One. 2023 Apr 13;18(4):e0282795. doi: 10.1371/journal.pone.0282795 (PMC10101483; doi:10.1371/journal.pone.0282795)
Supplement: S4 File — Odds ratios of obesity according to whether or not the cohort member had experienced multi-partner fertility and excluding childhood BMI. Baseline outcome: No multi-partner fertility. (DOCX) [file pone.0282795.s004.docx]

**Supplementary materials 4. Odds ratios of obesity according to whether or not the cohort member had experienced multi-partner fertility and excluding childhood BMI. Baseline outcome: no multi-partner fertility.**

|  | Fathers (n=3088) | | | Mothers (n=3532) | | |
| --- | --- | --- | --- | --- | --- | --- |
|  | Obesity | | | Obesity | | |
| MPF | OR | Sig. | 95% CI | OR | Sig. | 95% CI |
| Unadjusted association | 1.12 |  | (0.91, 1.38) | **1.31** | ****** | **(1.10, 1.56)** |
| (+) Parental controls^1^ | 1.00 |  | (0.81, 1.25) | 1.17 |  | (0.98, 1.41) |
| (+) Child controls age 10 – excluding child BMI^2^ | 0.95 |  | (0.76, 1.18) | 1.10 |  | (0.92, 1.33) |
| (+) Child controls age 16^3^ | 0.96 |  | (0.77, 1.20) | 1.11 |  | (0.93, 1.34) |
| (+) Adult controls age 42^4^ | 0.98 |  | (0.78, 1.22) | 1.03 |  | (0.85, 1.24) |

** P ≤ 0.05, ** P ≤ 0.01, *** P ≤ 0.001*

*^1^Parental education, parental social class, maternal age, maternal smoking; ^2^child cognitive ability, illness or disability, overcrowding, benefits; ^3^self-esteem, locus of control, malaise, Rutter behaviour, parental separation, smoking status; ^4^age at first birth, education, smoking, AUDIT, housing tenure, malaise.*
