# Supplementary material for: Exploring the associations between number of children, multi-partner fertility and risk of obesity at midlife: Findings from the 1970 British Cohort Study (BCS70)
Source: PLoS One. 2023 Apr 13;18(4):e0282795. doi: 10.1371/journal.pone.0282795 (PMC10101483; doi:10.1371/journal.pone.0282795)
Supplement: Attachment — Submitted filename: reviewPLOSONE.docx [file pone.0282795.s005.docx]

**Research article: Exploring the associations between number of children, multi-partner fertility and risk of obesity at midlife: Findings from the 1970 British Cohort Study (BCS70)**

**Review**

Generally, the manuscript was well written and the results were clearly presented.

1. Introduction was understandable, the individual paragraphs followed each other logically and clearly defined the importance and the need of the presented study.

2. An array of confounders which predispose individuals to obesity is very comprehensive.

3. A very extensive study sample ensures the accuracy of the results.

4. Statistical methods are suited to research questions posed at the study.

5. Results of the study are well presented and clearly explained.

6. Research question is clearly defined and appropriately answered.

Despite the above facts, I have a few following minor comments for the authors:

1. Various adult mediators at age 42 have been selected with the assumption that these affect obesity at age of 46. However, it is not clear from the article why the authors decided on the age of 42. Is there any scientific explanation for this selected age?
2. The authors revealed an important gender differences in the effect of parity as well as MPF on obesity manifestation at midlife. They correctly explained the reasons for this fact in the last paragraph of the section Discussion. However, some additional important data about female reproductive factors should be also considered and discussed. In the context with the recent findings that obesity at midlife is significantly associated not only with pregnancy characteristics, but also with other female reproductive factors, such as age at menarche and breastfeeding (Vorobelova et al. 2022, Ciesla et al. 2021). And thus, they present an important confounders influencing obesity at midlife.
3. In section „Strengths and limitations“ is written (lines 374-376): „Supplementary Materials S2 and S3 we compare the nested regression models testing the relationship between MPF and obesity for mothers and fathers including and excluding child BMI measured at age 10.“

However, neither in the methods nor in any other paragraph was there any information about the body mass index cutoff points for evaluation of nutritional status in 10 years old children. It is known that the cut off points differ between children and adults as established Cole et al. (2000). For example, the cut off point for BMI for obesity in 10-years old girls is 24,11 kg/m^2^. Therefore, I recommend to add the reference into the text.

(Establishing a standard definition for child overweight and obesity worldwide: international survey, *BMJ* 2000; 320) doi: <https://doi.org/10.1136/bmj.320.7244.1240>

4. The year of publication is missing in the reference with the number 99 (Light A, Ahn T. Divorce as risky behavior. Demography. 47, 895–921).
